# Foreign

**Published:** 1842-01-01

**Authors:** 


### FOREIGN.

*Operations for Wry-Neck.*—The following case of wry-neck is taken from a paper by J. Nottingham, Esq., late House Surgeon to the Liverpool Infirmary. The paper also contains notes referring to three cases of division of the tendo Achilles in pes equinus complicated with injuries of the hip joint in childhood and permanent limitation of motion in that joint the club-foot; being, in each case, the consequence of the injury to the hip. As the simple statement of this complication contains all that is particularly interesting to the surgeon in the paper, we will merely add that two of the operations were speedily successful in restoring the foot to its proper position ; thus rendering the limb more useful. The results of division of the sterno-cleido-mastoid muscle, together with the character of the changes effected in the cervical vertebræ by their long continued mal-position in torti-collis, is a question on which we have fewer lights of experience ; and for this reason we give the case,—which was a mild one,—in the words of Mr. Nottingham.

Mary Sesnan, aged twelve years, has had wry-neck since she was twelve months old, for which the mother cannot assign any cause; the distortion is considerable, and she complains of inability to move the head with freedom.

The sterno-mastoid of the right side is in a state of permanent contraction; the right ear pulled down towards the corresponding shoulder; the chin twisted to the opposite side; the distance between the meatus of the ear and the top of the sternum is much less on the right side than on the other. It is the true sterno-mastoid, not the clavicular portion of the muscle, that is contracted.

A sharp-pointed narrow bistoury was passed under the affected muscle from within outwards, a little below the crossing of the omo-hyoideus tendon, its edge then directed towards the skin, and the sternal portion of the muscle divided. Its section was accompanied by a sensation communicated to the finger more or less like that the surgeon perceives when the tendo Achillis is cut across.

We immediately observed a difference in the position of the head, and on the affected side it was obvious that the distance between the top of the sternum and the meatus of the ear was about an inch and a half greater than before; but it was thought that further improvement might probably be effected by another division of the muscle near its attachment to the mastoid process, which was made by gliding the bistoury under the integument and cutting down upon the bone. The effect of this section was not so great as that produced by the first.

A little adhesive plaster was applied over the punctures, and two or three turns of a roller round the neck.

It is now five weeks since the muscle was divided; the head can be turned to either side with facility, and the distortion is much diminished; the cervical vertebræ, however, long accustomed to peculiar position, do not at once retain the improved direction, which, by a little artificial support, can be given to them. The patient expresses herself with satisfaction respecting the "easy" manner in which she can now move and hold her neck.

Since the report of the division of the sterno-mastoid was written, improvement has gradually taken place in the neck of the patient; and we have every reason to be satisfied with the results of the operation.—*London Lancet, Nov.* 6, 1841.

In this case we can see no propriety in the division of both extremities of the muscle.—Thus far, the case was one of hyper-operation. Both heads should have been divided below, and at once, or the superior attachment should have been alone involved.

*Post Mortem examination after a case of an Crural Aneurism, cured by the constant application of Ice.*—A man named Gloria was attacked with aneurism, involving the crural and the external iliac. After being badly treated, the disease being mistaken, for many months, he entered the Marine Hospital, at Toulon, the tumor seeming likely to burst every moment. This aneurism was cured by the application of ice, and the man, who seemed at the point of death, was restored, after a course of treatment of nearly three years, to perfect health. He returned to duty on ship-board, and made a voyage to the West Indies: his health was perfect; the tumour had disappeared; and though the leg was rather larger than the other, yet motion was not painful; in short, he was fit for duty on ship-board. Towards the end of 1838 he was seized with pain in the pelvis and right leg, fever, &c.; a profuse suppuration and an enormous abscess resulted; it opened spontaneously, and discharged very freely; the patient broke down under the profuse discharge, and died in February, 1839.

On examination, post mortem, the right thigh was found larger than the left, and its cellular tissue, as well as that of the pelvis, infiltrated with a flaky serum, *(un serum couenneuse.)* Several large collections of pus were found in the internal iliac fossa, the sheath of the psoas, and in the thigh, from Poupart's ligament to the middle. The muscles pale, and infiltrated with pus. The femur and the vertebræ, though denuded of periostium at particular points, were not diseased. The external iliac artery was little enlarged, but the epigastric, the circumflex Ilii, and the subcutaneous abdominis, were considerably dilated. The crural artery, from the origin of the last named artery to that of the profunda, was entirely obliterated, transformed into a fibrous cord, the fibres of which were in the axis of the artery. A little above the origin of the profunda was a tumor half an inch in diameter, dense and fibrous in structure, quite distinct from the cellular tissue around it. This tumor was united to the artery by a small pedicle, at the upper part of which was an ossific point about a line in diameter. This autopsy confirms the case, (before published,) and shows the power of this remedy even in cases nearly desperate. In this case, a sac so thin that its giving way was hourly to be looked for, gradually felt the astringent and tonic power of the steady application of cold; and all that remained of the once formidable disease, was the small fibrous cord and little tumor, which did not, for three years, interfere with the man's performing the laborious duties of his station on ship-board.—"*New York Gazette,*" *from Gazette Medicale.*

---

*The Medical Examiner* is published every Saturday by *J. C. Turnpenny*, N. E. corner of Spruce and Tenth streets. Terms—*three dollars* a year for one copy, or *five dollars* for two copies sent to the same post office, in all cases in advance. Postage the same as on newspapers. Letters to be addressed,—post-paid,—to the Editors of the Medical Examiner, Philadelphia.
*Reynell Coates, M. D.*, Acting Editor.
*J. B. Biddle, M. D.*, and *W. W. Gerhard, M. D.*, Co-editors.
*J. B. Biddle, M. D.* Proprietor.

